# Roles of NK Cell Receptors 2B4 (CD244), CS1 (CD319), and LLT1 (CLEC2D) in Cancer

**DOI:** 10.3390/cancers12071755

**Published:** 2020-07-01

**Authors:** Casey W. Buller, Porunelloor A. Mathew, Stephen O. Mathew

**Affiliations:** Department of Microbiology, Immunology and Genetics, University of North Texas Health Science Center, Fort Worth, TX 76107, USA; Casey.Buller@my.unthsc.edu (C.W.B.); porunelloor.mathew@unthsc.edu (P.A.M.)

**Keywords:** natural killer (NK) cells, 2B4, CS1, LLT1, cancer, immunotherapy

## Abstract

Natural killer (NK) cells play a pivotal role in the immune system, especially in the recognition and clearance of cancer cells and infected cells. Their effector function is controlled by a delicate balance between the activating and inhibitory signals. We have identified 2B4 (CD244, SLAMF4) and CS1 (CD319, SLAMF7) as NK cell receptors regulating NK cell cytotoxicity. Lectin-like transcript 1 (LLT1), a member of the C-type lectin-like domain family 2 (CLEC2D), induced IFN-γ production but did not directly regulate cytolytic activity. Interestingly, LLT1 expressed on other cells acts as a ligand for an NK cell inhibitory receptor NKRP1A (CD161) and inhibits NK cytolytic function. Extensive research has been done on novel therapies that target these receptors to increase the effector function of NK cells. The 2B4 receptor is involved in the rejection of melanoma cells in mice. Empliciti, an FDA-approved monoclonal antibody, explicitly targets the CS1 receptor and enhances the NK cell cytotoxicity against multiple myeloma cells. Our studies revealed that LLT1 is expressed on prostate cancer and triple-negative breast cancer cells and allows them to evade NK-cell-mediated killing. In this review, we describe NK cell receptors 2B4, CS1, and LLT1 and their potential in targeting cancer cells for NK-cell-mediated immunotherapy. New cancer immunotherapies like chimeric antigen receptor T (CAR-T) and NK (CAR-NK) cells are showing great promise in the treatment of cancer, and CAR cells specific to these receptors would be an attractive therapeutic option.

## 1. Introduction

Cancer remains a prevalent disease throughout the world and is a prolific area of active research. Cancer is categorized as metastatic and nonmetastatic, with metastatic cancer being the leading cause of death in cancer patients [1]. A typical response from the immune system results in apoptosis of cancer cells [2]. Instead, cancer cells have a way of evading the immune response and undergoing further proliferation. The American Cancer Society projects that in 2020 there will be 1,806,590 new cancer cases and 606,520 cancer deaths in the United States [1]. Although much progress has been made in overcoming this disease, there is still much to learn about the progression of cancer and how it can be better targeted for therapy. Conventional therapies include the use of chemotherapy and radiation, but alternatives such as immunotherapy and the use of non-chemotherapeutic drugs are being researched. Conventional therapies are nonspecific as they kill cancer and healthy cells which could be very damaging to the individual as it can cause them to be in an immunosuppressive state whereby recurrent infections can occur [3,4]. Also, the use of conventional therapies creates the possibility of further inducing mutations in cancer and noncancer cells [4,5,6]. The use of alternatives to chemotherapy and radiation offers benefit to individuals affected by cancer, as it decreases toxic side effects. Additionally, the use of immunotherapies is intriguing because it can induce memory function of the adaptive immune system, leading to future clearance in recurring cancer [7]. It is also more tolerable for the individual due to immune tolerance mechanisms established by the immune system [7].

## 2. Immune Cells Involved in Immunosurveillance

Innate and adaptive immune cells are involved in the response to cancer cells. Most notably, natural killer cells and CD8^+^ T cells play an integral role in the clearance of immunogenic cancer cells. These cells have a cytotoxic effect and are good at eliminating the strongly immunogenic cancer cells, whereby they make way for the proliferation of less immunogenic cancer cells. Other immune cells that are involved in cancer progression are macrophages, neutrophils, dendritic cells (DC), and B cells [2]. Macrophages progress from proinflammatory (“M1” type) to anti-inflammatory (“M2” type) cells [2,8]. Proinflammatory macrophages aid in the elimination of cancer cells, but as they progress to an anti-inflammatory cell, they become more protumorigenic [8]. A similar process of specific proinflammatory and anti-inflammatory tumor-associated neutrophils is thought to occur, but distinct populations of neutrophils have yet to be characterized [9]. Dendritic cells play an important role in initiating the adaptive immune response. It has been shown that secretion of certain proteins into the tumor microenvironment impairs the recruitment of dendritic cells [10]. B cells are present in some cancers, but their role is not well understood [2]. Compelling evidence suggests that B cells are protumor in nature [11,12]. There are still other mechanisms by which immune cells play a pivotal role in the progression of cancer cells. 

### Evasion of the Immune System by Cancer

Cancer cells can evade the immune system by multiple mechanisms, but they stem from two main categories: avoiding immune recognition and nurturing an immunosuppressive tumor microenvironment [2]. Cancer cells can shed or downregulate major histocompatibility complex class I (MHC-I) molecules, thereby masking themselves from CD8+ T cells [13,14]. Initially, cancer cells express MHC-I complexes because they are “self”, and all nucleated cells have an MHC-I complex for CD8+ T cell recognition [14]. Due to selective pressures, cancer cells shed MHC-I molecules so that an adaptive immune response is no longer present. The transition from expressing MHC-I (“permissive” phase I) to no longer expressing MHC-I molecules (“nonpermissive” phase II) is due to different molecular mechanisms [15]. MHC-I complexes can be salvaged with the help of Th1 cytokine secretion, but if genetic defects occur to the heavy chain or β-2-microglobulin (β2M), then it can be irreversible [14,15,16]. In addition, cancer cells can cause the upregulation or downregulation of cell surface receptors or their ligands that allows them to camouflage themselves from natural killer (NK) cells [17,18].

The tumor microenvironment (TME) is composed of different infiltrating host cells and different molecules that are secreted from cancer cells [19,20]. One mechanism that tumor cells use that is well characterized is the programmed cell death protein 1 (PD-1)/PD-ligand (PD-L) pathway. PD-1 is expressed on T cells [21]. PD-1 can also be expressed on B cells, natural killer T cells, monocytes, and dendritic cells, but not resting T cells [22]. PD-ligand has two different splice variants, PD-L1 and PD-L2 [23]. PD-L1 is expressed on various immune cells and tissues but can also be secreted in a soluble form [23,24]. Tumor cells can secrete PD-L1 or upregulate secretion of PD-L1, which acts as an inhibitory signal to PD-1 [21]. It does not directly contribute to cell death but is involved in the downregulation of effector T cell function and antigen tolerance [22]. Therefore, tumor cells can inhibit the effector function of T cells by secreting inhibitory proteins leading to the suppression of the immune response in the TME. TME also provides molecules like TME-derived transforming growth factor-β (TGFβ) that downregulate NK cell cytotoxicity, cytokine secretion, metabolism, and proliferation, while inducing effector NK cells to upregulate innate lymphoid cell (ILC1)-like characteristics. Matrix metalloproteinases (MMPs) and a disintegrin and metalloproteinases (ADAMs) can shed receptors from the tumor cell surface, impeding the activation of NK cells and leading to ineffective effector functions [25].

## 3. Natural Killer Cells

Natural killer (NK) cells are innate lymphoid cells that play a major role in antitumor and antiviral responses. They are derived from hematopoietic stem cells and mature from the common lymphoid progenitor (CLP) cells. They function within the innate immune system and can be cytotoxic. NK cells were previously classified as group 1 innate lymphoid cells (ILC1), but recent evidence shows that ILC1 is a distinct subpopulation of lymphoid cells that are tissue-resident cells [26]. Although they both differentiate from the innate lymphoid common progenitor (ILCP), they express different transcription factors that separate them. Another defining difference is that ILC1 is tissue-resident, while NK cells are in the systemic circulation [26,27]. Therefore, NK cells can be classified as CD3- CD56+ [28]. Even this classification requires further specification. There are two subpopulations of CD56^+^ NK cell populations. These are CD56^bright^ and CD56^dim^ cells. The bright cells specialize in cytokine secretion, while the dim cells take on a more cytotoxic function through the secretion of IFN-γ, perforin, and granzyme [28].

NK cell effector function closely resembles that of cytotoxic T cells, but NK cells do not require prior antigen exposure to elicit their effector functions [29]. NK cells require a delicate balance of inhibitory and activating signals to elicit their effector functions. Their effector functions include cytotoxicity, antibody-dependent cell-mediated cytotoxicity (ADCC), cytokine and chemokine secretion [30,31]. NK cells reside in the systemic circulation until they are recruited into the peripheral tissues. Still, they need to be activated to elicit their full effects. They can be activated by type I interferons or proinflammatory cytokines such as interleukin 12, 15, or 18 [30,32]. 

### 3.1. Cellular Mechanisms of NK Cell Cytotoxicity

NK cell cytolytic function occurs through complex cellular processes which can be divided into three main processes: (1) target cell recognition, (2) target cell contact and immune synapse (IS) formation, and (3) NK-cell-induced target cell death. NK cells store contents such as granulysin, perforin, and granzyme. For these contents to be exocytosed to target cells, they must travel along the microtubules of the cell. Cytotoxic granules first converge at the microtubule-organizing center (MTOC), where they are transported to a dense F-actin network at the periphery of the cell. The F-actin network is traversed by lytic-granule-associated nonmuscle actin motor myosin IIa. Other accessory proteins such as UNC-45a—a chaperone protein—and Rab27a—a small GTPase—stabilize the myosin IIa and help the granule travel near the immune synapse. After traversing the F-actin network, the lytic granule must dock and tether to the inner portion of the plasma membrane so that it can exit the cell into the immune synapse via exocytosis. Tethering occurs through interaction between Rab27a and Munc13-4, which allows the lytic granule to bind to the inner plasma membrane. Priming occurs through the release of endoplasmic reticulum Ca^2+^ into the cytoplasm via hydrolysis of phosphatidylinositol 4,5-bisphosphate (PIP_2_) to diacylglycerol and inositol 1,4,5-trisphosphate (IP_3_) by phospholipase C-gamma (PLCγ). IP_3_ is released into the cytoplasm, where it can bind to Ca^2+^ receptors on the ER. The increased intracellular Ca^2+^ concentration is sensed by the C2 domains of the Munc13-4 protein. This causes the lytic granules to be primed and fused with the cellular membrane to release their contents into the immune synapse of the target cell via the interaction of various vesicle (v-SNAREs) and plasma membrane (t-SNAREs) proteins [33].

### 3.2. Role of NK Cells in Cancer

NK cells express a potent cytolytic function towards infected or aberrant cells [34]. NK cells are unique in that they can recognize the expression of MHC-I complexes on cancer cells. If there is downregulation of MHC-I complexes, then NK cells can target those cells for cell death, whereas the normal expression of MHC-I on healthy cells sends an inhibitory signal to NK cells [35]. The delicate balance between inhibitory and activating signals tailors the effector function of NK cells. Several inhibitory receptors that recognize MHC-I molecule, such as the killer cell immunoglobulin-like receptor (KIR) and leukocyte immunoglobulin-like receptor (LILR) families in humans, the Ly49 family in mice, and the CD94/NKG2 receptor family found both in mice and humans, and non-MHC-I-recognizing receptors such as T cell immunoglobulin and ITIM domain (TIGIT); carcinoembryonic Ag cell adhesion molecule 1 (CEACAM1), killer cell lectin-like receptor G1 (KLRG1), NKR-P1(A/B), sialic-acid-binding immunoglobulin-like lectin (Siglec), the Tyro3, Axl, and MerTK (TAM) receptors have been found to play a role in tumor immune surveillance [36]. On the other hand, activating receptors like CD16, NKG2D, NKp46 (natural cytotoxicity receptor 1 (NCR1)), DNAM-1, 2B4, NTB-A, and CS1 have been found to be critical for tumor surveillance in both human and murine NK cells [36,37]. NK cells have been well documented as being present in specific cancer types such as colorectal and gastric tumors [2,38]. Monalizumab, a humanized anti-NKG2A antibody, promoted NK cell and T cell activity against various tumors in mice and humans [39]. A recent report showed that STING-activating cyclic dinucleotides (CDNs) induced NK cell activation, cytotoxicity, and antitumor responses in an interferon-dependent manner in various tumor models [40]. Several obstacles must be overcome to utilize NK cells in cancer therapy; including blocking the inhibitory receptors that dampen NK cell activation; eliminating regulatory T cells (Treg) that suppress their function; allowing NK cells to traffic into solid tumors; neutralizing immunosuppressive cytokines such as TGFβ secreted by the tumor; eradicating myeloid-derived suppressor cells (MDSCs); and providing essential growth factors and cytokines that are required for NK cell activation, proliferation, and persistence. Strategies to resolve these issues are rapidly being developed and may enhance NK-cell-based therapies in the near future [41]. We have extensively studied the roles of 2B4, CS1, and lectin-like transcript 1 (LLT1) in targeting cancer cells for NK-cell-mediated killing; therefore, the main focus of this review is on these three receptors. 

## 4. NK Cell Receptors

### 4.1. Characterization of the 2B4 Receptor

**Figure 1:**
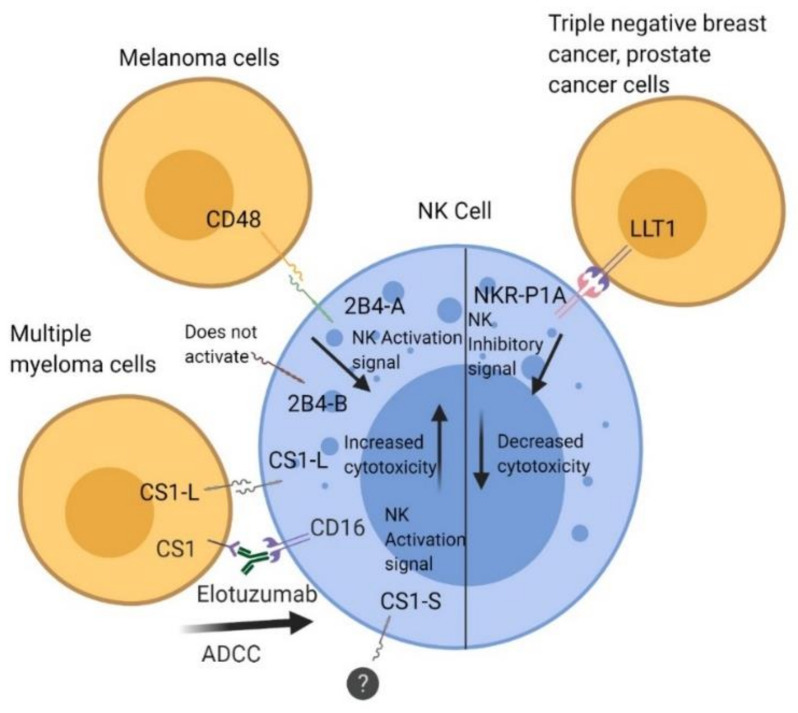
Interaction of 2B4, CS1, and lectin-like transcript 1 (LLT1) with their ligands in regulation of natural killer (NK) cell function. The 2B4 receptor is a heterophilic receptor that interacts with CD48 (SLAMF2) and has an activating function that increases cytolytic function in NK cells. CS1 is a homophilic receptor and comes in two isoforms: CS1-L and CS1-S. CS1-L has an activating role that improves cytolytic function, whereas CS1-S has no known function, but both are expressed constitutively on NK cells. Antibody-dependent cell-mediated cytotoxicity (ADCC) occurs through binding of the Fc portion of Elotuzumab to CD16 receptors on NK cells. LLT1 is a heterophilic receptor that interacts with NKR-P1A on NK cells. Interaction of LLT1 on triple-negative breast cancer (TNBC) and prostate cancer cells with NKRP1A on NK cells inhibits cytolytic activity.

The 2B4 receptor (SLAMF4, CD244) is a member of the signaling lymphocyte activation molecule (SLAM), which is a part of the immunoglobulin (Ig) superfamily [42,43,44,45]. SLAM family receptors are expressed on a repertoire of immune cells that are primarily involved in NK cell activation. The 2B4 receptor is expressed on both specific cell subsets and various tissues, including NK cells, a subset of CD8 T cells, γδ-T cells, monocytes, basophils, eosinophils, the spleen, lymph nodes, and numerous other tissues [42,46]. It has an N-terminal extracellular domain, variable-type Ig-like domain (IgV), constant two-type Ig-like domain (IgC2), a single transmembrane domain, and a cytoplasmic tail containing three tyrosine motifs (in human 2B4) [45,47]. The tyrosine motifs follow a TIYxxV/I (T: threonine, I: isoleucine, Y: tyrosine, x: any amino acid, V: valine) pattern that contributes to SLAM-associated protein (SAP) adaptor binding [47]. SLAM-related receptors are typically homophilic, but 2B4 interacts with a separate CD48 ligand [47,48,49]. CD48 is a glycophosphatidylinositol (GPI)-linked member of the CD2 family and is expressed on all hematopoietic cells [42,50]. Studies have shown that cytotoxicity occurs through the release of cytolytic granules containing perforin and granzymes [32,51,52]. Interestingly, a number of studies have indicated an activating role for 2B4 in humans [52,53], whereas it was shown to be inhibitory in mice [54,55,56]. To carry out its natural signaling function, the 2B4–CD48 interaction needs a SLAM-associated protein (SAP) adaptor [57]. Humans with X-linked lymphoproliferative disease 1 (XLP1) show an impaired function of SAP that causes an inhibitory signal rather than an activating signal to occur [57,58]. Loss of function of SAP can decrease the cytotoxicity of NK cells, thereby impairing its direct killing of tumor or virally infected cells. In mice, 2B4 has two isoforms: a long form (2B4-L), with four tyrosine motifs in the cytoplasmic domain, and a short form (2B4-S), with only one tyrosine motif. Functional studies in a rat NK cell line (RNK-16) transfected with 2B4-L and 2B4-S showed that 2B4-S activated while 2B4-L inhibited cytotoxicity of RNK-16 cells [59]. We have also shown that stimulation of NK cells through surface 2B4 downregulates its own expression due to a reduction in the promoter activity at the Ets element. The downregulation of 2B4 could be a mechanism to attenuate the co-stimulatory signal from 2B4–CD48 interactions [60]. We have determined that human 2B4 has two functional isoforms: h2B4-A and h2B4-B [42]. h2B4-A elicits a stronger cytotoxic function and intracellular calcium release compared to h2B4-B (Figure 1). h2B4-B has an additional five amino acids in the extracellular region of the receptor [42]. Interestingly, upon prolonged stimulation of 2B4, h2B4-A and h2B4-B are downregulated and their surface expression is decreased, suggesting a negative feedback loop that occurs to fine-tune the response of NK cell effector function [42].

The signaling pathway of 2B4 is not fully understood; rather, it is a complex pathway that may involve multiple adaptor molecules like linkers for activation of T cells (LAT), Ras, and Raf, which in turn activates p38 and MEK1/ERK mitogen activated protein kinase (MAPK) pathways [61]. The cytoplasmic tail of 2B4 contains immunoreceptor tyrosine-based switch motifs (ITSMs) which bind to SAP and send activation signals. In the absence of SAP, SHP1/SHP2 or EAT2 binds to the ITSM and mediates the inhibitory signal [62]. Our previous work showed that the isoform phosphokinase C-delta (PKC-δ) contributes to IFN-γ production and could also play an important role in the activation of activator protein-1 (AP-1) [63]. Interestingly, the activation of 2B4 shows a different response depending upon which cell model is being used. For instance, in murine NK cells, stimulation of 2B4 with an anti-CD244 mAb lead to increased IFN-γ production and increased non-MHC-restricted killing of tumor cells [64,65]. It was shown that the CD244–CD48 interaction is instrumental in proliferation of activated NK cells by IL-2. In human NK cell models, there is a different outcome. Stimulation of human NK cells by anti-CD244 mAb showed to have the same enhanced cytotoxic ability against target cells but had an antagonistic effect on the IL-2-stimulated proliferation of NK cells [65,66]. This experimental finding suggests that there is some other mechanism at play that contributes to the difference in outcomes. A previous study indeed showed that the expression level of 2B4 on NK cells could be causing this effect that is dependent on the CD244:SAP expression ratio [65].

### 4.2. Role of 2B4 and CD48 in Cancer

In order to understand the in vivo role of 2B4, 2B4-deficient mice were generated, allowing a complex role of 2B4 in rejecting B16 melanoma cells. Transfected CD48^+^ B16 melanoma cells were poorly rejected by wild-type (WT) mice, suggesting that the expression of CD48 on tumor cells inhibits the killing of B16 cells. This response is normal, as the 2B4–CD48 interaction is involved in an inhibitory response in mice but elicits an activating response in humans. Interestingly, female mice that were 2B4^−/−^ did not have any improvement in controlling tumor growth compared to their male counterparts, revealing a gender-specific role of 2B4 which was independent of CD48 expression on tumor cells [67]. It is suggested that neighboring T cells that express the CD48 receptor could help modulate an intermediary function, but also likely are two distinct mechanisms: the interaction of NK-cell 2B4 with tumor-associated CD48 and the 2B4–CD48 interactions among NK cells [56,67]. Lymphokine-activated killer cells (LAK) from WT and knockout male and female mice showed similar cytotoxicity against the B16 cell line, indicating that NK cells from female mice do not have an intrinsic defect. In vivo studies showed that there was no sex difference between NK-depleted mice and B16 tumor proliferation, suggesting that the gender differences observed are due to NK cells [67]. Put together, the studies with ex vivo LAK cells and in vivo NK-depleted mice indicate the possibility of a non-NK-cell role that is dependent on NK cell interaction with other cells. Furthermore, the possibility of this being driven by the neighboring T cells is supported by the interaction between 2B4 on NK cells and CD48 on T cells and its ability to enhance T cell proliferation [68].

The 2B4 receptor also appears to predominantly display inhibitory signaling in tumor-associated immune cells, but the factors that determine activating versus inhibitory signaling depend on the cell surface density of 2B4 and decreased or absent concentrations of SAP [65]. Zhang et al. reported that 2B4 has a differential effect on leukemia-initiating cells (LIC) as compared to normal hematopoietic stem cells (HSC). They further showed that 2B4 may co-operate with c-Kit to mediate its downstream signaling through SHP-2/p27 to regulate the proliferation (self-renewal) of LICs [69]. In glioma patients, CD48 was correlated with malignant progression, poor prognosis, immunosuppression, and inflammatory responses [70]. Alloferon, an immunomodulatory peptide, has been shown to increase NK cell cytotoxicity and antiviral effects. Increased NK cell natural cytotoxicity against tumor cells induced by alloferon was mediated by the upregulation of 2B4. It was also observed that alloferon caused increased IFN-γ and TNF-α production and granule exocytosis in NK cells [71]. Chimeric antigen receptor T (CAR-T) and NK (CAR-NK) cell therapies are the use of T and NK cells that have been engineered to express a specific receptor that can target a particular protein [72,73]. A CAR-NK cell containing the transmembrane domain of NKG2D, the 2B4 co-stimulatory domain, and the CD3ζ signaling domain significantly inhibited tumor growth and prolonged survival compared with peripheral blood NK cells, induced pluripotent stem cell (iPSC) NK cells, or CAR-T-iPSC-NK cells in an ovarian cancer xenograft model [74].

### 4.3. Characterization of the CS1 Receptor

CS1 (SLAMF7, CD319, CRACC) was initially identified, cloned, and characterized in NK cells, but now it has been found to play a central role in other immune cell functions. CS1 is located along the long arm of chromosome 1 (1q23.3) and is a part of the CD2 subset of the immunoglobulin superfamily of receptors [75]. Like its other close SLAM family receptors, CS1 contains unique tyrosine motifs (TxYxxV/I) along with its cytoplasmic domain and is homophilic [44,76]. Typically, the SLAM family receptors need the SAP adaptor to initiate downstream signaling from its immunoreceptor tyrosine-based switch motifs (ITSMs), but CS1 is unique in that it does not require SAP to transmit downstream signaling [77]. CS1 can recruit Ewing’s sarcoma’s/FLI1-activated transcript 2 (EAT-2), which is a SH2-domain-containing protein similar to SAP that phosphorylates tyrosine moieties in the cytoplasmic region [78]. This ultimately leads to cytotoxicity upon the activation of downstream effectors, including PLCγ and PI3K [78,79]. Interestingly, in humans, CS1 has two splice variants, namely a long-form CS1-L and a short form CS1-S. Both of these splice variants are constitutively expressed on NK cells; the CS1-L isoform has ITSMs, whereas CS1-S does not. CS1-L acts as an activating receptor, whereas CS1-S does not activate NK cells (Figure 1) [77]. In mice, transcription of CS1 is regulated by Yin Yang 1 (YY1) and a unique (AG)_36_ DNA repeat element. It is thought that YY1 acts as a transcriptional repressor since transcription of CS1 increased when YY1 was mutated [80]. In humans, the promoter region of CS1 contains a B-lymphocyte-induced maturation protein-1/positive regulatory domain zinc finger protein 1 (Blimp-1/PRDM1) binding site that has a trans-activating function in CS1 gene regulation, and when Blimp-1/PRDM1 is mutated, its transcriptional ability is decreased [81].

### 4.4. Targeting CS1 with a Monoclonal Antibody

CS1 expression in multiple myeloma (MM) has been extensively studied. Multiple myeloma (MM) is a disease of malignant plasma cells in the bone marrow characterized by anemia, lytic bone lesions, and elevated M protein in blood or urine and is associated with renal dysfunction [82]. CS1 was found to be overexpressed in plasma cells and has a high expression in MM cells [83,84]. Since CS1 is homophilic and is expressed on both NK cells and plasma cells, it was prudent to target CS1 as a novel immunotherapy for MM patients. This targeted therapy was first investigated in 2008 using HuLuc63, a humanized anti-CS1 mAb. Elotuzumab (Empliciti) was shown to inhibit adhesion of MM cells to bone marrow stromal cells (BMSC) and induce antibody-dependent cell-mediated cytotoxicity (ADCC) via NK cells [83]. Co-treatment of elotuzumab with bortezomib, a proteasome inhibitor, showed promising results of inhibition of tumor growth in the OPM2 xenograft model [85]. Later studies with combinations of elotuzumab, lenalidomide, and dexamethasone implied that elotuzumab alone produces low concentrations of IFN-γ and TNF-α [86,87]. Treatment with both elotuzumab and lenalidomide showed increased levels of secreted IFN-γ and TNF-α, with TNF-α directly contributing to enhanced NK cell cytotoxicity. IL-2 was also present and determined to be emitted from a subset of lymphocytes that are CD3+/CD56+ [86]. Two proposed mechanisms suggest that NK cells have effector function on MM cells through ADCC and NK activation with enhanced cytolytic function. ADCC occurs through binding of the Fc portion of elotuzumab to CD16 receptors on NK cells. NK cell activation and enhanced cytotoxicity occurs through elotuzumab stimulation of CS1 receptors on the NK cell surface, which leads to cytotoxicity [31,86,88,89]. Phase III trials were carried out through 2015, and elotuzumab was subsequently approved by the FDA to use in combination with lenalidomide and dexamethasone [90].

### 4.5. Targeting CS1 for CAR-T and NK Cell Therapy

Although much of the focus has been on CAR-T immunotherapy treatment in the past, CAR-NK therapy is also gaining some traction. Currently, there are several CAR-T therapies targeting SLAMF7 that have shown great promise in preclinical and clinical studies [91]. Compared to CAR-T cell treatment, CAR-NK cell treatment offers several advantages, but each of them offers exciting possibilities for the treatment of cancer [92,93]. Several reports have indicated the efficacy of CAR-T cells against B lineage cancers, but they have been associated with serious complications like cytokine release syndrome (CRS) and neurotoxicity. Allogenic cord blood CAR-NK cells were found to display favorable clinical responses without any CRS or neurotoxicity [94]. Chu et al. reported the use of a CS1-specific CAR-NK cell treatment for multiple myeloma. NK-92 and NKL cell lines were used to adequately express a CS1 construct of PCDH lentiviral vector backbone, sequentially containing a signal peptide (SP), CS1-specific scFV, a Myc tag, a hinge, CD28, and CD3ζ. Co-culturing the NK-92-CAR and NKL-CAR cells with different strains of multiple myeloma cells showed increased IFN-γ in both cell lines, but the concentration of IFN-γ (in pg/mL) was higher in the NK-92-CAR cell line. This also corresponded with a direct increase in cytotoxicity that was dependent on CS1 expression. Similarly, NK-92-CAR was tested ex vivo and in a xenograft model, which showed promising results [92]. This study now adds to the vast tools that could be potential players in the treatment of MM, either as a monotherapy or as a combination treatment with a current therapy for MM. As stated previously, CAR-T cell treatments continue to dominate research (as of April 2020, there are 92 CAR-T cell trials for MM treatment, compared to 1 CAR-NK cell clinical trial (NCT03940833)), but there is a great need to explore CAR-NK cell treatments as well. 

### 4.6. Characterization of the LLT1 Receptor

Lectin-like transcript 1 (LLT1), or osteoclast inhibitory lectin (OCIL), is a member of the C-type lectin-like domain family 2 (CLEC2D) subfamily [95]. LLT1 is part of a larger superfamily of proteins called C-type lectin-like domains (CTLDs) and is present on NK cells, T cells, monocytes, macrophages, and activated dendritic and B cells. Upon its initial discovery, the term CTLD was meant to signify a member of the group of Ca^2+^-dependent (C-type) carbohydrate-binding proteins that bind to carbohydrates, Ca^2+^, and other molecules. All CTLDs have a characteristic double-loop structure that forms antiparallel beta-sheets. Additionally, multiple disulfide bridges formed by cysteine residues help keep the structure together [96]. CTLDs have a wide variety of functions, including homeostatic and antimicrobial functions [97]. LLT1 was first identified in 1999 on the human NK gene complex with proximity to CD69 along chromosome 12 [95]. In NK cells, when LLT1 is cross-linked with an anti-LLT1 monoclonal antibody, it induces NK cells to produce IFN-γ without any increase in cytotoxic function [98]. Later research showed that LLT1 expressed on target cells is the ligand for the NKR-P1A (CD161) receptor on NK cells and inhibits NK cell cytotoxicity. NKR-P1A is also expressed on subsets of T cells. Interaction between LLT1 and NKR-P1A induced IFN-γ secretion through TCR signaling [99,100]. Although the function of NKR-P1A-LLT1 has been elucidated, the signaling by which it occurs is still not understood well. Thus far, our lab has shown that LLT1 signaling pathways are likely dependent upon Src protein tyrosine kinase (Src-PTK), p38, and ERK signaling pathways, by which ERK signaling is associated with IFN-γ production [101].

### 4.7. LLT1 Expression in Cancer

**Figure 2:**
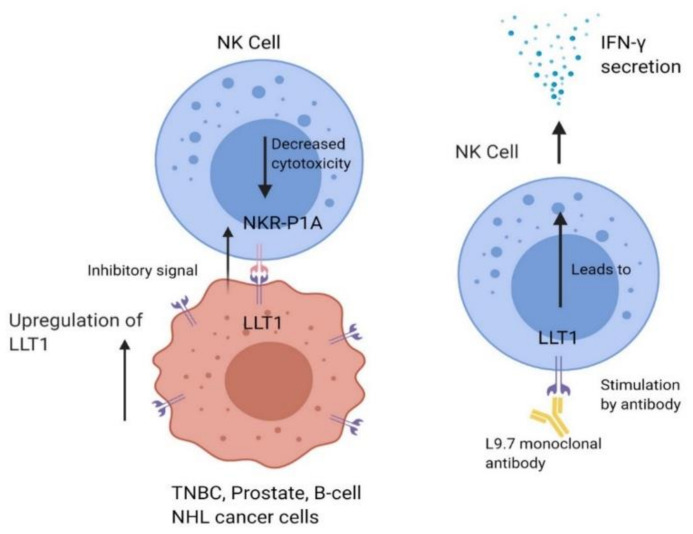
LLT1 is expressed on specific tumor cells such as triple-negative breast cancer (TNBC), prostate cancer, and B-cell non-Hodgkin’s lymphoma (NHL) cells. LLT1 on tumor cells interacts with NKR-P1A on NK cells, sending an inhibitory signal to NK cells which decreases cytolytic function. Cancer cells have also been shown to upregulate LLT1 expression to increase the immune dampening effect.

Because LLT1 can be expressed on different cell and tissue types, it presents the possibility for the cancer cells to escape the immunosurveillance of NK cells [102]. We have shown that the expression of LLT1 on target tissues such as triple-negative breast cancer cells and prostate cancer cells inhibits the NK cell response (Figure 2) [17,103]. 

Additionally, expression of LLT1 on germinal-center-derived B cell non-Hodgkin’s lymphoma has also been shown to have a dampening effect on the function of NK cells [104]. Increased expression of LLT1 implies that cancer cells actively upregulate LLT1 expression, but the mechanism by which this occurs is unknown. Interestingly, expression of LLT1 and CD161 in lung cancer is associated with a better clinical outcome. One primary difference is that a distinct NK cell population was not observed in non-small-cell lung cancer (NSCLC). Instead, a distinct CD161+, CD4+, and CD8+ T cell population was present within the tertiary lymphoid structures [105]. Although this evidence is different from previously established evidence that LLT1/CD161 acts as an inhibitory signal, this highlights the difference between the innate and adaptive immune response.

This suggests that LLT1 has a dual function but is dependent on the immune cell type that expresses it. This evidence contributes to the growing knowledge of LLT1 and suggests that it could potentially be a good target for novel therapies.

## 5. Tumor Microenvironment

### Tumor Microenvironment’s Effect on NK Cell Function

The tumor microenvironment (TME) can have a profound effect on the ability of immune cells to carry out their effector function. Multiple immune cell types are associated with various types of tumors and their TME. These include tumor-associated macrophages (TAMs), myeloid-derived suppressor cells (MDSCs), regulatory T cells (Treg cells), neutrophils, and NK cells, amongst various others [106]. TME leads to NK cell suppression through different mechanisms and evasion through tumor cell immune-editing [106,107]. Suppression can occur through secretion of certain cytokines like TGFβ, which downregulates NK cell receptors NKp30 and NKG2D [108]. Both receptors are critical for tumor recognition and cytotoxic killing. Previous research shows that patients who have a gastrointestinal stromal tumor (GIST) have increased Treg cells present within the TME, and secretion of TGFβ is correlated with decreased NK cell activity [109,110]. Additionally, downregulation of the NKG2D ligand on the tumor cell surface contributes to tumor cell immune-editing [111]. Although there has been no apparent research done to determine how the TME affects 2B4, CS1, and LLT1 receptors specifically on NK cells, there has been some research done concerning the effect of the TME on 2B4 in T cells. Recently, researchers showed that cholesterol concentration increased with increased expression of PD-1+ and 2B4+ infiltrating CD8+ T cells in patients with colon cancer and myeloma. Furthermore, the increased expression of PD-1+ and 2B4+ correlated with increased exhaustion of T cells due to increased cholesterol uptake from the surrounding TME [112]. The tumor cell is a complex structure, but the TME, with the involvement of various cell types that affect immune cell effector functions, may prove to be even more complex to decipher. 

## 6. Conclusions

NK cells play a prominent role in the innate immune system’s antitumor and antiviral response. NK cells have a multitude of known receptors that interact with cancer cells. Utilizing the ability of NK cells to target cancer cells is something that future therapies need to employ, as NK cell therapy can be effective if adequately harnessed. We have cloned three receptors, namely 2B4, CS1, and LLT1, that have shown to play important roles in the effector function of NK cells against cancer. CAR-NK cells containing NKG2D and 2B4 domains have shown promising results in ovarian cancer xenograft studies. On the other hand, monoclonal antibody therapy Empliciti targeting CS1 has been very effective in the treatment of multiple myeloma. CS1 represents a viable target for CS1-specific CAR-NK cells to treat multiple myeloma. The expression of LLT1 on several different cancer tissues and its interaction with CD161 on NK cells provides a way for cancer to escape the immunosurveillance function of NK cells by inhibiting its cytolytic activity. Novel therapies targeting these receptors show great promise in cancer immunotherapeutics, but more research needs to be done to establish the safety and efficacy of these treatments. In a broader context, this new research could lead to enhancing mAb or checkpoint inhibitor treatments that are already being tested in other types of cancer [39,40,113]. Another consideration in future studies is the recent findings in NK cell tumor immunology with respect to soluble secreted ligands and any effects on 2B4, CS1, and LLT1 [114,115]. A better understanding of the functions of 2B4, CS1, and LLT1 in different cancers will usher new treatment options, especially for those that do not respond to conventional therapies.
